# 3D Printed Skin‐Interfaced UV‐Visible Hybrid Photodetectors

**DOI:** 10.1002/advs.202201275

**Published:** 2022-07-11

**Authors:** Xia Ouyang, Ruitao Su, Daniel Wai Hou Ng, Guebum Han, David R. Pearson, Michael C. McAlpine

**Affiliations:** ^1^ Department of Mechanical Engineering University of Minnesota Minneapolis MN 55455 USA; ^2^ Department of Dermatology University of Minnesota Minneapolis MN 55455 USA; ^3^ Sino‐German College of Intelligent Manufacturing Shenzhen Technology University Shenzhen 518118 P. R. China

**Keywords:** 3D printing, fully 3D printed electronics, hybrid organic‐inorganic materials, photodetectors, wearable electronics

## Abstract

Photodetectors that are intimately interfaced with human skin and measure real‐time optical irradiance are appealing in the medical profiling of photosensitive diseases. Developing compliant devices for this purpose requires the fabrication of photodetectors with ultraviolet (UV)‐enhanced broadband photoresponse and high mechanical flexibility, to ensure precise irradiance measurements across the spectral band critical to dermatological health when directly applied onto curved skin surfaces. Here, a fully 3D printed flexible UV‐visible photodetector array is reported that incorporates a hybrid organic‐inorganic material system and is integrated with a custom‐built portable console to continuously monitor broadband irradiance in‐situ. The active materials are formulated by doping polymeric photoactive materials with zinc oxide nanoparticles in order to improve the UV photoresponse and trigger a photomultiplication (PM) effect. The ability of a stand‐alone skin‐interfaced light intensity monitoring system to detect natural irradiance within the wavelength range of 310–650 nm for nearly 24 h is demonstrated.

## Graphical abstract

A flexible UV‐vis photodetector array that incorporates a hybrid organic‐inorganic material system is fully 3D printed. The hybrid active material enables a photoresponse in the UV region and triggers a photomultiplication (PM) effect. The printed device is integrated with a console and functions as a stand‐alone skin‐interfaced light intensity monitoring system, which has potential for the medical profiling of photosensitive cutaneous diseases.

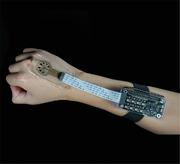

## 1 Introduction

Wearable and skin‐interfaced electronic devices that continuously monitor environmental signals in situ and thereby serve as real‐time health‐profiling strategies have the potential for mitigating the severity of environmentally‐sensitive diseases.^[^ ^1^ ^]^ Some skin diseases, such as lupus erythematosus (LE), an autoimmune disorder with characteristic skin and systemic manifestations, may be triggered or exacerbated via UV exposure from the sun or even ambient indoor light.^[^ ^2^ ^]^ Broad‐spectrum environmental light exposure, particularly in the ultraviolet B (UVB) band covering the spectral range of 280 to 310 nm and the ultraviolet A (UVA) band spanning the spectral range of 310–400 nm, exerts a variety of clinical repercussions in LE patients.^[^ ^3^ ^]^ Accordingly, there is a need for a skin‐interfaced photodetector system that quantitatively measures irradiance across clinically relevant spectral bands, in order to assess disease‐exacerbating exposures in situ.^[^ ^4^ ^]^ With advancements in miniaturized photodetector and device integration technologies, commercially available UV photodetectors have been successfully incorporated into wearable UV‐monitoring devices for continuous health intervention technologies.^[^ ^5^ ^]^ Commercial silicon photodetectors have several advantages, including compatibility with silicon electronics and a low‐noise signal profile. Yet, such devices also face several important limitations, such as limited mechanical flexibility and weak absorption over a broadband spectrum which curtails either UV or visible sensitivity.^[^ ^6^ ^]^

Organic semiconductors, including small molecules and polymers, provide an ideal class of photoactive materials for compliant photodetectors due to their mechanical flexibility.^[^ ^7^ ^]^ Moreover, compared to silicon‐based counterparts, organic photodetectors provide additional advantages including solution‐based processing methods, tunable optoelectronic performance, more uniform affinity to the target surface, and lower cost.^[^ ^8^ ^]^ However, inorganic photodiodes have high sensitivities due to the avalanche effect or impact ionization, while photodetectors comprising only organic photoactive materials have limited capacities to effectively detect weak light signals. This is a result of the charge generation yields being affected by the larger exciton binding energies and disordered molecular stacking of organic semiconductor materials.^[^ ^9^ ^]^ This limited capability diminishes the clinical application of these devices for photosensitive LE patients, since incident UV light is less intense than visible light due to absorption by ozone, water vapor, and other molecules in the air.^[^ ^10^ ^]^ Therefore, amplification of the photocurrent by the photomultiplication (PM) effect is vital to enhance the photoresponse of the organic photodetectors, which can be achieved by introducing trap states for charge tunneling injection.^[^ ^11^ ^]^ With this approach, multiple charge carriers can be gathered when one incident photon is absorbed, resulting in external quantum efficiencies (EQE) which can exceed 100%.^[^ ^12^ ^]^ Zinc oxide (ZnO) is a suitable candidate as a carrier trap material, which can trap electrons due to local defects on the ZnO surface.^[^ ^13^ ^]^ ZnO nanoparticles (NPs), a widely‐used low‐cost metal‐oxide in commercial sunscreens,^[^ ^14^ ^]^ can be deployed in UV photodetectors due to their wide direct bandgap.^[^ ^15^ ^]^ As a result, the organic semiconductor material can be doped with the inorganic ZnO NPs to formulate a hybrid active material that enhances the photoresponse and extends the spectral region to the UV range.^[^ ^16^ ^]^ To date, most studies have concentrated on performance improvements of the photodiodes under a negative bias voltage greater than −1.5 V, which is the standard voltage of a battery. For a wearable device to efficiently function in a low‐power and electrically safe state, it is necessary to optimize the hybrid active materials to achieve a high EQE under low bias voltage.

Three‐dimensional (3D) printing technologies can fabricate devices from a broad palette of materials, without requiring conventional fabrication techniques such as spin‐coating, templates, photolithography, or high vacuum metal deposition.^[^ ^17^ ^]^ Among currently available 3D printing technologies such as inkjet printing,^[^ ^18^ ^]^ aerosol jet printing,^[^ ^19^ ^]^ optical printing,^[^ ^20^ ^]^ and powder bed fusion‐based printing,^[^ ^21^ ^]^ extrusion‐based 3D printing can accommodate a broad range of printable viscosities of multi‐functional inks.^[^ ^22^ ^]^ Furthermore, extrusion‐based 3D printing is suitable for fully 3D printed functional devices using a “multi‐scale” printing approach, incorporating nanoscale inks printed at the micron scale to fabricate macro‐scale devices,^[^ ^23^ ^]^ and the integration of multiple functionalities on rigid or flexible substrates,^[^ ^24^ ^]^ or even on moving objects.^[^ ^25^ ^]^

A flexible, stretchable, and biocompatible substrate is essential for a fully 3D printed wearable and skin‐interfaced device to allow the device to interact with the human body and the environment naturally and safely.^[^ ^26^ ^]^ Polydimethylsiloxane (PDMS) is commonly used for this type of elastic substrate to achieve biointerfaced compliance.^[^ ^27^ ^]^ However, achieving uniform wetting of the PDMS film with functional inks is challenging due to the low surface energy of PDMS.^[^ ^28^ ^]^ Therefore, surface modification methods such as UV‐ozone (UVO) treatment, plasma treatment, or other chemical coatings are typically employed to regulate the wettability of the PDMS substrate,^[^ ^29^ ^]^ which helps to precisely define the pattern and layout of the electrodes and active components of the device.^[^ ^30^ ^]^ The flexible and functional devices may then be 3D printed on the PDMS substrate and transferred to human skin.

Here we introduce a fully 3D printed flexible hybrid UV‐visible (UV‐vis) photodetector array fabricated on a stretchable substrate, which is then integrated with a custom‐built portable console for continuous long‐term monitoring of light intensity. Compared with our previously reported polymer photodetectors,^[^ ^31^ ^]^ the active material is doped with ZnO NPs, and the surface roughness and optical‐transmission characteristics of the hybrid active layer are studied. The optimized formulation of the hybrid active material improves the performance of the photodetector by extending the spectral response to the UV band and enhancing the photoresponse via the PM effect. The 3D printed hybrid photodetector demonstrated a responsivity of 0.51 A W^–1^ and an EQE of >100% at 310 nm at a bias of as low as −1 V, suitable for battery‐operated wearable devices. The flexible photodetectors, printed on PDMS films, demonstrated reliable performance stability during both optical and mechanical tests. The 3D printed photodetector array was then incorporated with eight optical bandpass filters of different central wavelengths ranging from the UVB to the visible band. The incorporated photodetector array was integrated with a custom‐built Python‐based console for a compact skin‐interfaced light intensity monitoring system, demonstrating the capability to continuously detect light intensity under natural sunlight over an extended time period.

## 2 Results and Discussion

### 2.1 Design of the Light Intensity Monitoring System

**Figure 1:**
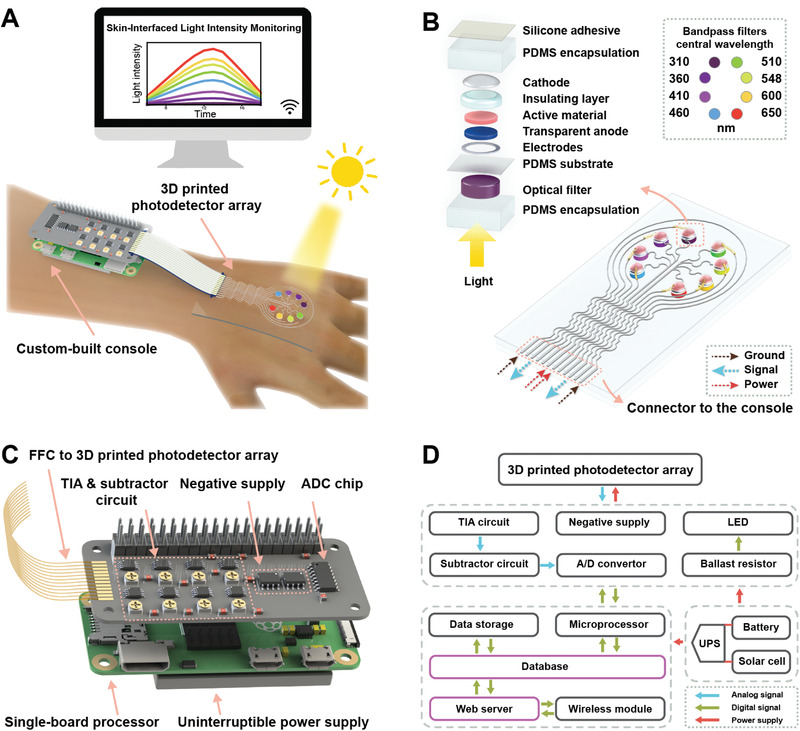
Skin‐interfaced photodetector system for in‐situ light intensity monitoring. A) Schematic illustration of the photodetector array with a console in operation. The system consists of a photodetector array for monitoring the light intensity of eight different wavelengths and a custom‐built console for signal processing and wireless data transmission. B) Schematic of the 3D printed photodetector array. The photodetectors and optical filters with eight different central wavelengths are assembled on the top and backside of the flexible polydimethylsiloxane (PDMS) substrate, respectively. The left inset is an exploded view of one photodetector with an optical bandpass filter. The right inset is the positioning of the central wavelengths of the optical bandpass filters. C) Schematic of the console for the monitoring system. The console consists of a custom‐built signal processing board for driving photodetectors and processing photocurrent signals, a single‐board processor for data processing and transmission, and an uninterruptable power supply (UPS) module for power management. D) Schematic block diagram of the monitoring system.

The skin‐interfaced light intensity monitoring system (**Figure** **1**A) consists of a 3D printed UV‐vis photodetector array and a compact custom‐built console, which is flexible enough to be worn on the hand for in‐situ monitoring of light intensity. The eight‐channel photodetector array that converts light intensity into photocurrent signals is interfaced with the multi‐functional console via a flat flexible cable (FFC). The console supplies a bias of −1 V to the photodetector array to yield the photocurrent signals which are stored and plotted by the console. With an embedded Wi‐Fi module in the console, the eight‐channel light intensity data and charts can be wirelessly transmitted to computers and accessible via a web browser for real‐time monitoring.

The 3D printed eight‐channel photodetector array (Figure 1B) consisted of eight UV‐vis broadband photodetectors with different optical bandpass filters that define the specific spectral ranges of the photodetectors. Eight different optical bandpass filters, with central wavelengths ranging from 310 to 650 nm (Figure 1B right inset), were placed on the device side that received the incident light. In the circuit design of the photodetector array, each photodetector was connected to one individual signal line, and four photodetectors shared one common power line as a group. A protective grounding line enclosed the photodetector array to reduce the external electromagnetic interference to the device. Thus, twelve pins were used in total as connectors to the console. The serpentine shape of the electrodes was chosen in accordance with the design rules in stretchable electronics,^[^ ^32^ ^]^ which aid in enduring the increased tensile strain that occurs from movement‐induced deformation of the wearable device. After the printing was completed, the device was encapsulated by PDMS, which improved the mechanical stability and protected it from chemicals and moisture in daily use. A biocompatible silicone adhesive was coated on the PDMS encapsulation to adhere the photodetector array to the skin.

As shown in the exploded view of the photodetector (Figure 1B left inset), the incident light successively passes through the transparent PDMS encapsulation, an optical filter, the transparent PDMS substrate, and the circular transparent window defined by the silver electrode. Then, the filtered light propagates through the transparent anode layer that is printed with poly(3,4‐ethylenedioxythiophene):polystyrene sulfonate (PEDOT:PSS), and excites the hybrid active material (Figure S1, Supporting Information), a ternary mixture of poly(3‐hexylthiophene) (P3HT):[6,6]phenyl C61‐butyric acid methyl ester (PCBM):ZnO NPs. The energy levels in the diagram of the device (Figure S1, Supporting Information) were estimated from the literatures.^[^ ^12^, ^31^ ^]^ The active materials then absorb incoming photons and produce excitons that diffuse in the hybrid materials and disintegrate as free charge carriers, including holes and electrons, at the polymer/polymer and polymer/NP interfaces. Charge carriers are collected by the PEDOT:PSS anode and liquid‐metal (eutectic gallium indium, EGaIn) cathode, which generates the photocurrent signal. Some electrons are trapped on the ZnO NPs instead of being collected by the cathode, which enhances the charge tunneling injection and triggers the PM effect, thereby amplifying the photocurrent signal and improving the photoresponse of the device.

The console of the light intensity monitoring system comprised a custom‐built signal processing board, a single board that houses the microprocessor, and an uninterruptible power supply (UPS) module (Figure 1C). A twelve‐pin FFC connected the photodetector array to the signal processing board, among which two power lines were connected to the two negative supply chips. The eight‐channel photocurrent signals were processed by the transimpedance amplifier (TIA) circuit (Figure 1D), which converted the current signals to voltage signals. The voltage signals were then processed by subtractor circuits and further amplified to improve the dynamic range. The amplified analog signals were converted by the analog/digital converter (ADC) to digital signals, which were further processed by the single‐board processor (Figure 1D bottom left dashed line frame). The signals were stored locally in a database on the single‐board processor that can be exported as readable files or viewed on a graphically interactive webpage via a wireless connection. A feedback signal triggered a light‐emitting diode (LED) via the ballast resistor on the signal processing board to indicate the status of the console when the photocurrent signals were processed. A UPS module was used to manage the power sources among a battery, a solar cell, and a direct current power source so that a stable power source could be continuously provided to the photodetector system for the real‐time monitoring of light intensity.

### 2.2 Hybrid Active Material

**Figure 2:**
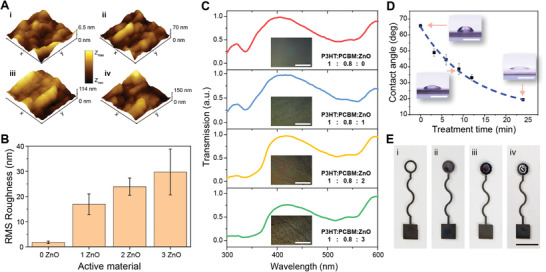
Characterization of the hybrid active materials and 3D printing of the photodetector. A) Atomic force microscopy (AFM) images of active films with 0ZnO (i), 1ZnO (ii), 2ZnO (iii), and 3ZnO (iv). B) Root mean square (RMS) roughness of films printed with the active materials (*n* = 5). C) Normalized transmission spectra of active films. The insets are optical microscope images of active films. The scale bars are 50 µm. D) The dependence of contact angle on UV ozone treatment time (*n* = 5). The insets are images of droplets on the PDMS film. The scale bars are 1 mm. E) Images of printing steps of one photodetector. The scale bar is 5 mm. In B) and D), data are presented as mean ± SD.

The organic active materials, P3HT and PCBM, have bandgaps of ≈1.9 eV and ≈2.2 eV,^[^ ^33^ ^]^ respectively, and have previously been 3D printed as a bulk heterojunction (BHJ) layer to detect light in the near UV to visible band.^[^ ^31^ ^]^ To increase the light sensitivity in the UV range, we added ZnO NPs as UV absorbers (bandgap = ≈3.4 eV) into the active materials.^[^ ^12b^ ^]^ As the photoresponse of the photodetectors is sensitively affected by the composition of the active material, we examined the effect of various weight ratios on the device performance with four different recipes: 1) P3HT:PCBM:ZnO = 1:0.8:0 [0ZnO], 2) P3HT:PCBM:ZnO = 1:0.8:1 [1ZnO], 3) P3HT:PCBM:ZnO = 1:0.8:2 [2ZnO], and 4) P3HT:PCBM:ZnO = 1:0.8:3 [3ZnO]. The rheological properties of the active materials were also characterized (Figure S3, Supporting Information). The viscosities of the active materials marginally rose as the shear rate increased. The slight increment in the viscosities could result from the development of secondary flow, i.e., Taylor–Couette flow, in the liquid as the shear rate increased, which is observed in low viscosity liquids.^[^ ^34^ ^]^ The atomic force microscopy (AFM) images (**Figure** **2**A) of the active layers (1 × 1 µm) revealed that the ZnO NPs were doped into the organic materials, and the surface roughness increased as the weight ratio of ZnO NPs in the hybrid materials increased (Figure 2B). Specifically, the root mean square (RMS) roughness of the photoactive layers printed with the recipes of 0ZnO, 1ZnO, 2ZnO, and 3ZnO were 1.80, 16.95, 23.92, and 29.74 nm, respectively. The increase in the RMS roughness was induced by the large particle size of ZnO relative to the organic molecules and particle aggregation as the weight ratio of ZnO increased.

We further characterized the optical transmission of the printed active films with the testing system (Figure S2, Supporting Information). Due to the wide bandgap, ZnO NPs showed a strong UV absorption from 300 nm to 375 nm (Figure S4A, Supporting Information). The normalized transmission spectrum of the active film with 0ZnO exhibited a small transmission peak and a wide transmission peak at ≈315 nm and ≈400 nm (Figure 2C), respectively. The small transmission peak at 315 nm decreased as the concentration of ZnO NPs in the active materials increased and remained at a low level in both 2ZnO and 3ZnO films, which indicated a strong light absorption in the UVB band. One can also observe a soft cutoff of ≈375 nm in the transmission spectra of 2ZnO and 3ZnO (Figure 2C), which was consistent with the transmission spectrum of ZnO NPs. The optical microscope image of the 3ZnO film showed that the nanoparticle aggregation results in a visible microscale roughness of the active film. Compared with the active film of 2ZnO, 3ZnO had a lower transmission between 400 and 560 nm, which may be attributable to the larger scattering and reflection of the rougher active film.

PDMS was chosen as the substrate for the 3D printed photodetectors because of its high transparency in the UV band (Figure S4B, Supporting Information). The widely used polyethylene terephthalate (PET) is unsuitable for this UV‐sensitive application due to its strong light absorption between 300 and 340 nm. The UVO treatment, which can cause polymer chain scission and result in polar chemical groups such as Si‐OH on the surface of PDMS,^[^ ^35^ ^]^ was used to improve the wettability of the PDMS substrate prior to printing the silver electrodes. The contact angle was measured to assess the wettability (Figure 2D). The solvent of the silver ink, triethylene glycol monoethyl ether, was used in the characterization of the contact angle. As the UVO treatment period increased from 0 to 24 min, the contact angle decreased from 65.72° to 19.35°, indicating improved wetting of the silver ink solvent on the PDMS surface. This is essential for precise 3D printing of electrodes, because an untreated surface with low surface energy causes the ink to form a series of discrete droplets instead of a continuous line. Based on our tests, PDMS surfaces with a long 24 min UVO treatment exhibited an overly small contact angle and prevented the ink from forming precise shapes. As a result, PDMS substrates with a 9 min UVO‐treatment were used in the printing of photodetectors.

With the ability to co‐deposit multiple functional materials, 3D printing was used to fabricate the hybrid photodetectors (Figure 2E, Figure S5, Supporting Information). The materials were 3D printed layer‐by‐layer on the UVO‐treated PDMS films. Printed silver electrodes with a width of ≈500 µm and thickness of ≈2.14 µm (Figure S4C, Supporting Information) exhibited a resistivity of 1.04 × 10^–6^ Ωm. The circular transparent window of the silver electrode (Figure 2E(i)) was designed to transmit the incident light to the photosensitive layer. The transparent conductive polymer PEDOT:PSS was then printed within the circular window of the silver electrode as the anode of the photodetector. Due to the electron‐blocking and hole‐transport properties of PEDOT:PSS, this anode layer helped to reduce the dark current of the photodetector.^[^ ^36^ ^]^ After thermal curing, the silver electrodes and PEDOT:PSS layers exhibited shrinkage of ≈0.43% and 5.11%, respectively. The ternary hybrid active material (Figure 2E(ii)) was then deposited on the PEDOT:PSS layer, and a ring‐shaped silicone insulation layer (Figure 2E‐iii) was subsequently printed on the photosensitive layer. Finally, the EGaIn liquid metal^[^ ^22^, ^37^ ^]^ (Figure 2E(iv)) was extruded on the predefined photosensitive area as the cathode layer. The insulation layer confined the shape and location of the liquid metal cathode and prevented it from displacing and contacting the silver electrode, avoiding short‐circuiting issues.

### 2.3 Photoresponse Performance

**Figure 3:**
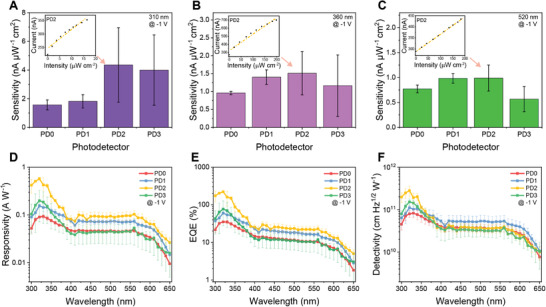
Characterization of photoresponse of printed photodetectors. A‐C) Sensitivity of photodetectors with varying active materials at a bias voltage of −1 V and wavelengths of A) 310 nm, B) 360 nm, and C) 520 nm, respectively (*n* = 3). The insets are current‐intensity characteristics of PD2. D‐F) Responsivity, external quantum efficiencies (EQE), and detectivity, respectively, at a bias voltage of ‐1 V of the photodetectors printed with the various active materials (*n* = 3). Data are presented as mean ± SD.

To determine the optimized photoresponse performance, the active material recipes, 0ZnO, 1ZnO, 2ZnO, and 3ZnO, were used to print the photodetectors, referenced as PD0, PD1, PD2, and PD3, respectively. A linear relationship was observed between the photocurrent and light intensity at a bias of −1 V under irradiation at 310, 360, and 520 nm (**Figure** **3**A–C insets, Figure S6, Supporting Information). The photocurrent and light intensity were then linearly fitted, and the slope of the fitted line was defined as the sensitivity. PD2 showed the highest sensitivities (Figure 3A–C) among the four types of photodetectors at 310, 360, and 520 nm, with values of 4.4, 1.5, and 0.99 nA µW^–1^ cm^2^, respectively. PD1, PD2, and PD3 showed higher sensitivity than PD0 at 310 nm (Figure 3A), which indicated that ZnO NPs, the UV absorber, increases the sensitivity at 310 nm. Compared with PD2, PD3 showed lower sensitivity at 310 and 360 nm (Figure 3A,B), which might result from the higher surface roughness of the active material with 3ZnO, which impacts the photoresponse performance by causing higher recombination rates.^[^ ^38^ ^]^ PD1 and PD2 showed higher sensitivities at 520 nm than PD0 (Figure 3C), even though the ZnO NPs increase surface roughness and do not exhibit a strong absorption in the visible band. The higher sensitivity is thus attributable to PM caused by ZnO NPs in the hybrid active materials.

The responsivity, which measures the electrical output per optical input, is a characteristic used to analyze the photoresponse of the photodiodes. The responsivity (*R*) of the photodetectors is given by^[^ ^39^ ^]^

(1) $$R=\frac{I_{\mathrm{light}}-I_{\mathrm{dark}}}{P_{\mathrm{irradiation}}}$$

in which *I*_light_ and *I*_dark_ are the currents under irradiation at a certain wavelength and in a dark environment, respectively, and *P*_irradiation_ represents the incident light power.^[^ ^39^ ^]^ The shapes of the responsivity spectra (Figure 3D) corresponded to the transmission spectra of the active layer, which indicated that the photoresponse originated from the hybrid active material. As shown in the responsivity of PD2, the broad peak of the responsivity spectra in the UV range (300–360 nm) and the dip at around 400 nm were consistent with the absorption peak in the UV range and transmission peak at around 400 nm, respectively. PD2 showed the highest responsivities among the photodetectors at 310, 360, and 520 nm, with values of 0.51, 0.2, and 0.09 A W^–1^, respectively. With a greater weight ratio of ZnO NPs with charge traps to trigger a substantial PM effect, PD2 showed enhanced responsivity compared to PD0 and PD1. Moreover, the active layer of PD2 had a lower surface roughness than PD3, leading to an improved photoresponse, which was consistent with the sensitivity characterization. These responsivity values in fully 3D printed hybrid photodetectors are comparable to those of commercial silicon‐based photodetectors (≈0.2 A W^–1^).^[^ ^40^ ^]^

The EQE is another representative performance indicator, which estimates the ability of the photodetectors to convert incident photons to current. The EQE is calculated according to,^[^ ^41^ ^]^

(2) $$\mathrm{EQE}=\frac{\mathrm{hcR}}{q\lambda }$$

where *R* is the responsivity, *λ* is the wavelength, *h* represents Planck's constant, *c* represents the speed of light, and *q* is the elementary electronic charge.^[^ ^41^ ^]^ The EQE curves (Figure 3E) were consistent with the transmission spectra of the active films and the responsivity of the photodetectors. The EQE of PD2 from 300 to 340 nm was above 100%, presumably due to the PM effect.^[^ ^11a^ ^]^ For PD0, the organic materials absorbed the incident photons and generated excitons (Figure S1, Supporting Information). The holes and electrons from photogenerated excitons moved from the highest occupied molecular orbital (HOMO) and lowest unoccupied molecular orbital (LUMO) of the active material, to the transparent PEDOT:PSS anode (Figure S1 Step 1, Supporting Information) and the EGaIn cathode through the PCBM layer (Figure S1 Step 2, Supporting Information), respectively. Due to the energy level difference, there was a larger injection barrier between the EGaIn (≈4.3 eV) and HOMO of the organic active materials (P3HT with ≈5.2 eV and PCBM with ≈6.1 eV), which prevented the holes from injecting into the active area. For the hybrid photodetector, the photogenerated electrons were trapped in the ZnO of the active layer (Figure S1 Step 3, Supporting Information) instead of directly transmitting to the cathode, because of the surface defects on the NPs.^[^ ^16^, ^42^ ^]^ The trapped electrons in ZnO NPs acted as space charges to produce a Coulomb field,^[^ ^11b^ ^]^ thereby inducing band bending near the cathode layer, which improved the hole tunneling injection by reducing the injection barrier (Figure S1 Step 4, Supporting Information) and therefore resulted in the PM effect.^[^ ^7^ ^]^ Subsequently, the holes from the external circuit were transmitted to the P3HT and transported together with photoinduced holes towards the PEDOT:PSS anode, which formed the enhanced photocurrent signal. As discussed previously, due to this hole tunneling injection, the EQE of photodetectors exhibiting the PM effect can be larger than 100%. Indeed, with a bias of −1 V, PD2 showed the highest EQE of 203.5% at 310 nm, 68.97% at 360 nm, and 22.18% at 520 nm among the four types of photodetectors. Because a higher bias voltage is needed for charge carriers to overcome the energy barrier, the EQE increased rapidly as the bias voltage increased by allowing more charge carriers to overcome the energy barrier (Figure S7, Supporting Information).

The specific detectivity (D*) describes the ability of the photodetectors to sense faint light intensities, and can be calculated and characterized using Equation (3) as^[^ ^31^ ^]^

(3) $$D^{*}=\frac{R\sqrt{A}}{\sqrt{2qI_{\mathrm{dark}}}}$$

where *R* is the responsivity, *A* is the active area of the photodetector, and *I*_dark_ is the dark current.^[^ ^31^ ^]^ The specific detectivities of PD2 were 2.49 × 10^11^, 9.21 × 10^10^, and 3.62 × 10^10^ cm Hz^1/2^ W^–1^ at 310, 360, and 520 nm, respectively. PD2 showed the highest specific detectivity in the spectral band ranging from 300 nm to 360 nm, while PD1 showed higher specific detectivity in the 370–650 nm range (Figure 3F). PD1 exhibited higher specific detectivity in the visible band than PD2 because PD1 showed similar responsivity, and the dark current was lower due to the smoother active films.

### 2.4 Mechanical and Electrical Stability of the Photodetectors

**Figure 4:**
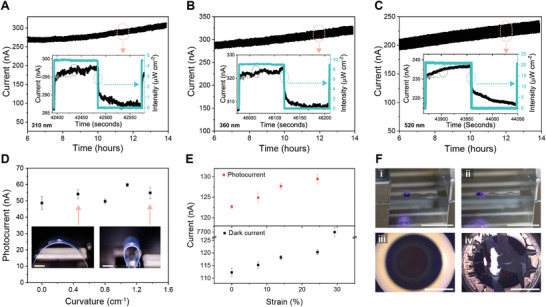
Stability and stretchability of the 3D printed photodetectors. A–C) Current response of a photodetector to 310 nm, 360 nm, and 520 nm on/off modulated light, respectively, in the last 8 h of a test period of 14 h. The light intensities used in A–C) are ≈4.6, 9.2, and 21.1 µW cm^–2^, respectively. The insets are magnified views of current responses. D) Bending test of the photodetector (*n* = 5). The left and right insets are the photodetectors undergoing curvatures of 0.46 and 1.37 cm^–1^, respectively. The scale bars are 10 mm. E) Photocurrent and dark current of the photodetector at varying tensile strains (*n* = 5). F) Images showing a photodetector under 0% (i) and 29.3% (ii) strain, respectively. The scale bars are 10 mm. (iii) and (iv) are optical microscope images of the sensing areas in (i) and (ii), respectively. The scale bars are 1 mm. In D) and E), data are presented as mean ± SD.

Due to its high performance in terms of sensitivity, responsivity, and EQE in the broadband, PD2 was selected for the wearable photodetector in the light intensity monitoring system. We investigated the electrical stability of PD2 under long‐term on/off modulated illumination (**Figure** **4**A–C, Figure S8, Supporting Information). Decays in the dark current and photocurrent were observed during the first 6 h of the electrical stability test period (Figure S8, Supporting Information). This reduction in current may result from the degradation of the polymer active material, which is common in organic optoelectronic devices.^[^ ^43^ ^]^ After 6 h, the current, including the dark current and photocurrent, increased over time, which might result from the persistent photoconduction phenomenon.^[^ ^44^ ^]^ This is caused by accumulated electrons in ZnO NPs due to the slow oxygen adsorption and desorption rate on ZnO surfaces.^[^ ^45^ ^]^ The response time (Figure 4A–C insets) included a fast transient (<0.1 s) followed by a slow rise (>10 s), which indicated that the photoresponse resulted from polymers and nanoparticle active materials.^[^ ^16a^ ^]^ The rise times of the photodetector under the illumination of 310, 360, and 520 nm were 34.9, 26.4, and 30.2 s, respectively.

The flexibility of the photodetector was characterized by attaching the device to a PET film and mounting it on a translation stage to adjust the bending curvature (Figure 4D insets). The bent photodetector was illuminated from the bottom side by a 405 nm laser diode. The light intensity of the laser was 47.46 µW cm^–2^. The photodetector showed stability under mechanical bending (Figure 4D) and cyclic bending tests (Figure S9, Supporting Information), showing no significant change in the photoresponse as the bending curvature changed due to the flexibility of the organic materials.

To characterize the stretchability, the photodetector was fixed on a one‐axis translation stage, and then strain was applied. The same laser diode was used in the stretchability test and the flexibility test. When strain increased on the photodetector, the photocurrent and the dark current increased (Figure 4E). This phenomenon might result from the proportional relationship between the sheet resistance of the active film and the thickness, which decreases under strain.^[^ ^46^ ^]^ Moreover, the change of reverse bias injection‐limited current (*I*) can be predicted by *I*∝*V*/*T*, with *V* as the voltage and *T* as the thickness of the layer.^[^ ^47^ ^]^ The proportion of *V* to *T* represents the effective electric field in the photosensitive layer, such that a larger effective electric field assists the charges to overcome the injection barrier and increase the current. The dark current at the strain of 29.3% experienced a sharp rise and reached a value of ≈7.53 µA because the active layer and transparent anode were damaged under the strain, through which the liquid metal cathode leaked into the anode and caused a short circuit (Figure 4F). The white spots in the sensing area showed liquid metal leakage, indicating short‐circuiting of the photodetector. As the transparent anode and active layer in the sensing area were vulnerable to tensile strain, the circular‐shaped electrode was designed for the sensing area to reduce the extension of the anode and active layer under tensile strain (Figure S10, Supporting Information). The cracks along the *y*‐axis (Figure S10 area 1, Supporting Information) in the central sensing area resulted from the stress along the *x*‐axis, while the wrinkles along the *x*‐axis in the silver electrode (Figure S10 area 2, Supporting Information) were caused by the stress along the y‐axis. The wrinkles also indicated that some parts of the electrode delaminated from the PDMS substrate during the tensile testing.

### 2.5 3D Printed Skin‐Interfaced Light Intensity Monitoring System

**Figure 5:**
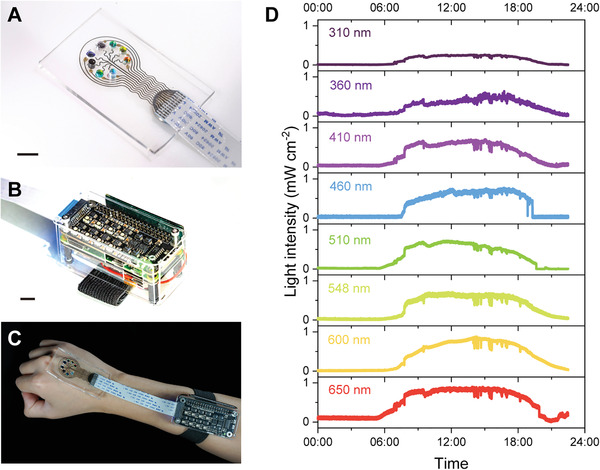
3D printed skin‐interfaced light intensity monitoring system. A) Image of the photodetector array for monitoring the light intensity of eight different wavelengths. B) Image of the console containing a signal processing board, a single‐board processor, and a UPS module. The console is connected to the 3D printed photodetector array via a flat flexible cable (FFC). The scale bars in figure A and B are 10mm. C) Image of the wearable photodetector system attached to a hand for in‐situ light intensity monitoring. D) The light intensity, measured by the monitoring system, of eight different wavelengths ranging from UVB to the visible band of natural sunlight during a full day in Minneapolis, Minnesota, on May 12, 2021.

The encapsulated 3D printed photodetector array (PD2) with eight optical filters (**Figure** **5**A) was connected to a customized electrical console (Figure 5B) via an FFC. The photodetector array was 3D printed layer‐by‐layer on the PDMS film (Figure S11, Movie S1, Supporting Information). The thicknesses of the anode layer, active layer, silicone insulation layer, and silver paste conductive interconnect were measured to be 288.43 ± 80.25 nm, 226.78 ± 74.85 nm, 60.41 ± 1.35 µm, and 42.22 ± 1.58 µm (*n* ≥ 3), respectively. The projected area of the console (Figure S12, Supporting Information) was smaller than a credit card, which made it viable to serve as a wearable device. The system could be powered by a lithium battery and charged by a solar cell or a commonly used 5 V charger due to the low bias voltage operation of the 3D printed hybrid photodetectors. By coating the device with a biocompatible silicone adhesive, the system could be firmly attached to the arm (Figure 5C, Movie S2, Supporting Information). The central wavelength of eight optical bandpass filters in the 3D printed photodetector array ranged from 310 to 650 nm (Figure S13, Supporting Information). In addition to the tests in the laboratory, we conducted an outdoor test consisting of recording the light intensity distribution for nearly 24 h in Minneapolis, Minnesota, on May 12, 2021, using the monitoring system. The system was exposed to natural sunlight and continuously recorded the light intensity with a time interval of ≈1 s. The light intensities (Figure 5D) generally increased after sunrise (≈06:00) and decreased gradually until sunset (≈20:30). The fluctuations of the distribution of light intensity after 12:00 correspond to passing cloud cover. A multifunctional web server with a graphical interface (Figure S13 and Movie S3, Supporting Information) was developed and executed on the monitoring system to allow facile access to the light intensity data. The figures for the light intensity distribution could be generated and downloaded via a wireless connection, rendering it convenient for physicians and users to continuously monitor environmental light exposure.

## 3 Conclusion

We have fully 3D printed hybrid UV‐vis photodetectors that were integrated with a portable custom‐built console for continuously monitoring light intensity as a stand‐alone skin‐interfaced device. The optimized hybrid active material, consisting of P3HT:PCBM and ZnO NPs, can trigger the PM effect and improve the photoresponse of the photodetectors over a broadband range, especially in the UV band. The EQE of the optimized photodetector exceeds 100% at 310 nm, and the responsivity at a bias of −1 V is 0.51 A W^–1^ which is on par with commercial‐grade silicon‐based photodetectors. The photodetectors printed on PDMS films exhibited high mechanical flexibility and stretchability, which is suitable for wearable devices. The eight‐channel photodetector array was then integrated with a custom‐built multifunctional console that supplied power to the photodetectors, processed the light intensity data, and delivered a graphical frontend for remotely accessing the light intensity distribution. The skin‐interfaced stand‐alone photodetector system possessed the ability to monitor outdoor broadband light intensity for nearly 24 h. In addition, this system continuously monitored in‐situ UV‐vis light intensity on the skin and recorded the long‐term light intensity distribution, which might serve as a suitable real‐time exposure‐monitoring strategy for photosensitive LE patients and assist in comprehensively analyzing UV‐enhanced broadband effects on other photosensitive skin diseases.

Future studies will involve: 1) developing wavelength‐tunable narrowband PM‐type photodetectors to eliminate the embedded optical filters; 2) further improving the detectivity by introducing an appropriate hole barrier layer;^[^ ^48^ ^]^ 3) extending the photoresponse to the near‐infrared region for phototherapeutic applications; 4) miniaturizing the photodetector system by reducing the feature sizes of the devices; 5) assessing the clinical acceptability of the monitoring system to target photoprotection strategies in LE and other photosensitive diseases; and 6) enhancing the flexibility and stretchability of the devices by engineering active and transparent anode layers and designing the mechanics‐guided serpentine‐shaped electrodes for the entire device.

## 4 Experimental Section

### Materials

P3HT (part number 900563), PCBM (part number 684430), ZnO NPs (part number 677450), EGaIn (part number 495425), silver paste (part number 735825), PEDOT:PSS solution (0.8 wt%, part number 739316), AgNPs dispersion (30‐35 wt%, part number 736465), trichloro(1*H*,1*H*,2*H*,2*H*‐perfluorooctyl)silane (TPFS, part number 448931), chlorobenzene (CB, part number 284513), 1,2‐dichlorobenzene (DCB, part number D56802), methanol (MeOH, part number 34860) were ordered from MilliporeSigma. Room temperature‐cure silicone (LOCTITE SI 595 CL) was ordered from Henkel AG & Co. Conductive epoxy (8331D‐14G) was ordered from MG Chemicals. PDMS (SYLGARD 184) was purchased from Dow Inc. Silicone adhesive (RT Gel 4317) was kindly provided by Elkem.

### Photoactive Ink Preparation

Solvent 1 resulted from mixing CB and DCB in a 9:1 weight ratio, and Solvent 2 was the mixture of MeOH and DCB in a 9:1 weight ratio. P3HT and PCBM solutions were dissolved in Solvent 1 with concentrations of 30 mg mL^–1^ and 24 mg mL^–1^, respectively. The photoactive material with an equal weight ratio of P3HT solution and PCBM solution was prepared. Then, the binary mixture was diluted with CB to 1/10^th^ of the initial concentration, and stirred at 700 rpm for 24 h. The ZnO NPs dispersion with a concentration of 30 mg mL^–1^ was produced by dispersing ZnO NPs in Solvent 2 followed by ultrasonication for 30 min and stirring at 900 rpm for 1 h. The average ZnO NPs size was ≈36 nm. To prepare 0ZnO, 1ZnO, 2ZnO, and 3ZnO, 0.15 mL Solvent 2; 0.1 mL Solvent 2 and 0.05 mL ZnO NPs dispersion; 0.05 mL Solvent 2 and 0.1 mL ZnO NPs dispersion; and 0.15 mL ZnO NPs dispersion were added into 1 mL diluted P3HT:PCBM solution, respectively. Next, the ternary active material was stirred at 700 rpm for 1 h.

### 3D Printing of Photodetectors

The glass substrate was cleaned by an ultrasonic bath in ethanol for 30 min. The UVO cleaner was used to treat the cleaned glass substrate for 20 min. The UVO‐cleaned glass substrate was then silanized with TPFS for a low surface‐energy coating,^[^ ^49^ ^]^ which was beneficial for peeling off the printed flexible photodetector array from the glass substrate. The treated glass substrate was placed in a vacuum chamber with a pump (DTC‐41, ULVAC KIKO Inc.), and a container with 50 µL TPFS was placed on the side of the glass. The reaction was conducted for 8 h, and then the silanized glass substrate was baked for 10 min at 80 °C. To produce the PDMS film, a blend containing the precursor and curing agent in a 10:1 weight ratio was made, defoamed in a mixer at 2200 rpm for 5 min, and then deposited on the silanized glass substrate by a spinner at 600 rpm for 1 min. Then, the PDMS films were baked at 70 °C for 3 h and treated by UVO for 9 min, modifying the surface for appropriate wettability. The translation stage (ANT130 Nanopositioning System, Aerotech) and pressure dispenser (Ultimus V, Nordson EFD) were used to print photodetectors on the treated PDMS film. The substrate was placed on the *X*‐*Y* translation stage, while a syringe with the appropriate nozzle (Nordson EFD) connected to the dispenser was mounted on the Z translation stage. Predesigned G‐code programs were used to control the printer and dispenser. The detailed printing parameters of each layer are summarized in Table S1, Supporting Information. From the literature,^[^ ^31^, ^50^ ^]^ the viscosities of the AgNPs dispersion, PEDOT:PSS and EGaIn are ≈15, 11, and 1.99 mPas, respectively. The two parts of conductive epoxy were blended in a weight ratio of 1:1 and then applied on the pins of the printed electrodes. Next, the FFC was attached to the pins with conductive epoxy, and a pair of magnetic pads assisted in fixing the FFC and the pins. The conductive epoxy was cured for 2 h at room temperature. Next, the optical filters were placed on the printed photodetector array. Subsequently, the device was encapsulated by PDMS and cured at 70 °C for 3 h. The silicone adhesive was coated on the encapsulated device. Finally, the 3D printed photodetector array was connected via the FFC.

### Device Characterization

In transmission measurements (Figure S2, Supporting Information), a Xenon lamp (L2174‐01, Hamamatsu Photonics) that emitted a broad continuous spectrum light was used as the light source. A filter wheel (FW1AND, Thorlabs) with neutral density filters (Thorlabs) was used to adjust the light intensity. The light was then focused and coupled into a bifurcated fiber bundle (BFY1000HS02, Thorlabs) using two UV‐fused silica lenses (Edmund Optics). The light from one output of the bifurcated fiber bundle illuminated the sample, and the transmission was measured by a UV‐vis spectrometer (FLAME‐S‐XR1‐ES, Ocean Insight). In photoresponse measurements, the light source was a broadband mercury lamp (S1500 without filters, OmniCure). The light from another output of the bifurcated fiber bundle was collimated and inputted into a monochromator (Cornerstone 130, Newport). The output light from the monochromator was coupled into a bifurcated fiber bundle, with one output light illuminating the sample and the other monitored by a UV‐extended photodiode power sensor (S130VC, Thorlabs, Inc.) connected to an optical power meter (PM100D, Thorlabs, Inc.). The photoresponse of the sample was then measured by a semiconductor device parameter analyzer (B1500A, Keysight). A manual one‐axis translational stage and a laser diode (CPS405, Thorlabs, Inc.) with neutral density filters were used in the flexibility and stretchability tests.

### Design and Fabrication of the Console

The signal processing board (Figure S12, Supporting Information) in the console contained eight amplification units, two negative supply units, and one ADC. The principles of the circuits can be found in the literature.^[^ ^51^ ^]^ As shown in the schematics of the amplification unit circuit, there were two operational amplifiers (OPAs) in one amplification chip (SM73307, Texas Instruments). The left OPA was used to build a TIA to convert the photocurrent to the voltage signal. Compared to an ordinary resistor for converting current to voltage, the TIA has advantages, including stable gain and a better signal‐to‐noise ratio. The amplification factor of the TIA was set by a feedback resistance network, i.e., a T‐type network. The converted voltage signal then was inputted into a subtractor circuit for subtracting a predefined constant which was close to the dark current signal of the photodetector. Next, the analog voltage signal was converted by an ADC (MCP3208, Microchip Technology) to a digital signal which could be transmitted to the Python‐based single‐board processor (Raspberry Pi Zero W) via a general‐purpose input/output (GPIO) port for further processing. Two CMOS switched‐capacitor voltage converters (TL7660, Texas Instruments) were used to perform positive‐to‐negative supply‐voltage conversions. The design of the negative supply was informed by the instruction of the datasheet of the chip. The resistors and capacitors of the circuits were ordered from Digi‐Key Electronics. The PCB boards were fabricated by PCBWay Technology.

In the single‐board processor, the serial peripheral interface ports on the GPIO header were used to communicate with the custom‐built signal processing board, and the inter‐integrated circuit ports were used to communicate with the UPS module (PiSugar2). The controlling program was based on Python. The graphic interface of the web page was generated by an open‐source Python library (Pyecharts). The web server of the console was based on the open‐source Apache Server, and the database server of the console was based on the open‐source MariaDB Server.

### Wearable Light Intensity Monitoring System

Xia Ouyang consented to wear the device during the experiments.

### Statistical Analysis

All experimental data including error bars are represented as the mean ± standard deviation. The sample size (*n*) for experimental data was included in the figure captions. All analyses were performed in OriginLab software.

## Conflict of Interest

M.C.M., D.R.P., X.O., and R.S. are inventors on a provisional patent application entitled “Photodetectors for measuring real‐time optical irradiance” related to this work filed on 13 June 2022, with the serial number 63/366,299. M.C.M. serves on the Scientific Advisory Board and holds equity in GRIP Molecular Technologies. M.C.M. is Co‐Founder and CSO of Flui3D Inc. D.R.P. is a consultant for Biogen Inc. and clinical trials principal/sub investigator for Corbus Pharmaceuticals, Elorac, Inc., Eli Lilly and Company, Emerald Health Pharmaceuticals, Kadmon, Inc., Pfizer, Inc., and Soligenix, Inc. These interests have been reviewed and managed by the U. of Minnesota in accordance with its Conflict of Interest policies.

## Supporting information

Supporting Information

Movie S1

Movie S2

Movie S3

## Data Availability

The data that support the findings of this study are openly available at The Data Repository for the University of Minnesota (https://doi.org/10.13020/10b2‐mp37).
