# Supplementary material for: 3D Printed Skin‐Interfaced UV‐Visible Hybrid Photodetectors
Source: Adv Sci (Weinh). 2022 Jul 11;9(25):2201275. doi: 10.1002/advs.202201275 (PMC9443467; doi:10.1002/advs.202201275)
Supplement: Supplementary file 1 — Supporting Information [file ADVS-9-2201275-s003.pdf]

ADVANCED SCIENCE
Open Access

Supporting Information

for *Adv. Sci.*, DOI 10.1002/advs.202201275

3D Printed Skin-Interfaced UV-Visible Hybrid Photodetectors

Xia Ouyang, Ruitao Su, Daniel Wai Hou Ng, Guebum Han, David R. Pearson and Michael C. McAlpine**

Supporting Information

3D Printed Skin-Interfaced UV-Visible Hybrid Photodetectors

Xia Ouyang, Ruitao Su, Daniel Wai Hou Ng, Guebum Han, David R. Pearson and Michael C. McAlpine**

X. Ouyang, R. Su, D.W.H. Ng, G. Han, M.C. McAlpine
Department of Mechanical Engineering, University of Minnesota, Minneapolis, MN 55455, USA.
E-mail: mcalpine@umn.edu

D.R. Pearson
Department of Dermatology, University of Minnesota, Minneapolis, MN 55455, USA.
E-mail: pearsond@umn.edu

X. Ouyang
Sino-German College of Intelligent Manufacturing, Shenzhen Technology University, Shenzhen, 518118, China

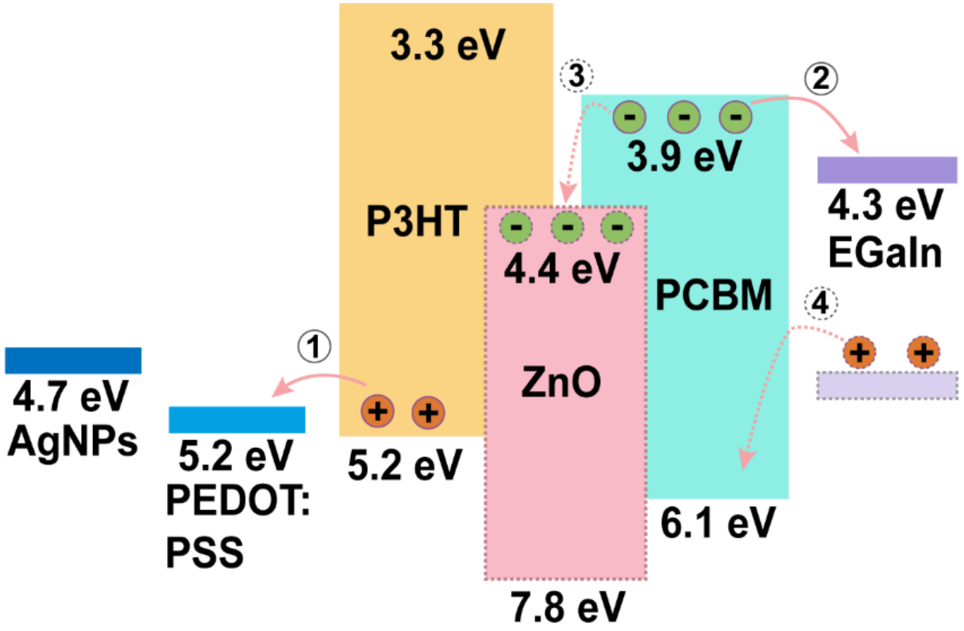

Figure S1. Energy diagram of the P3HT:PCBM:ZnO hybrid photodetector.[S1]

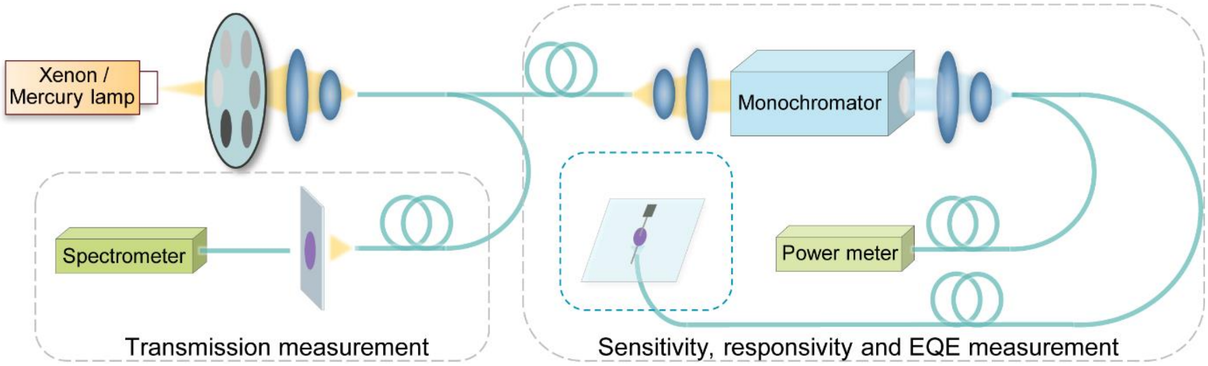

Figure S2. Schematic illustration of the testing setup.

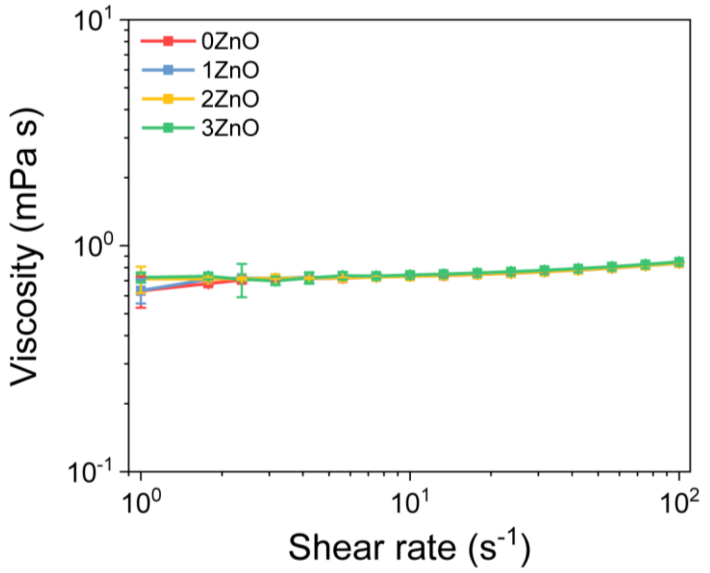

Figure S3. The rheological properties of the active materials (*n*=3), measured by a TA Instruments Discovery Series Hybrid Rheometer DHR-3. Data are presented as mean ± SD. Error bars are included but may not be visible for certain data points.

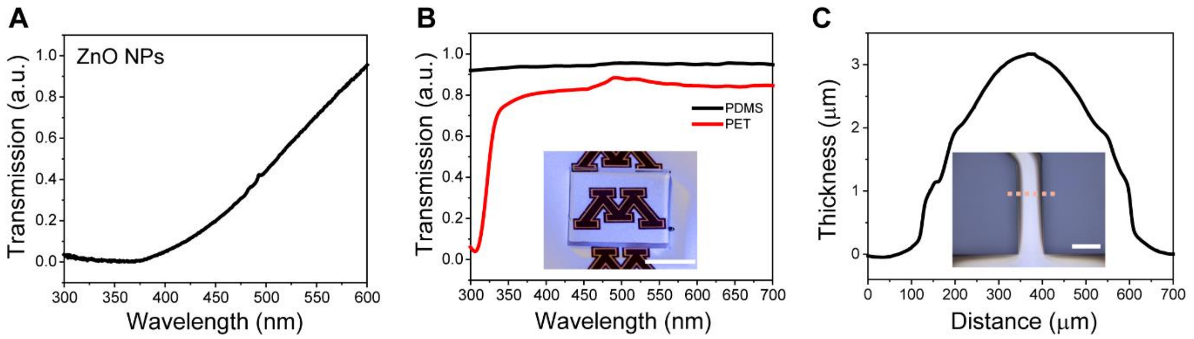

Figure S4. Characterization of the transmission and surface profile. A) Normalized transmission of ZnO NPs. B) Comparison of the transmission spectra of PDMS and PET films. The inset is a UV-transparent PDMS film. The scale bar is 1 cm. C) The cross-sectional profile of a printed silver electrode, measured by a Tencor-P7 Stylus Profiler. The inset is an optical microscope image of the silver electrode. The scale bar is 500 μm.

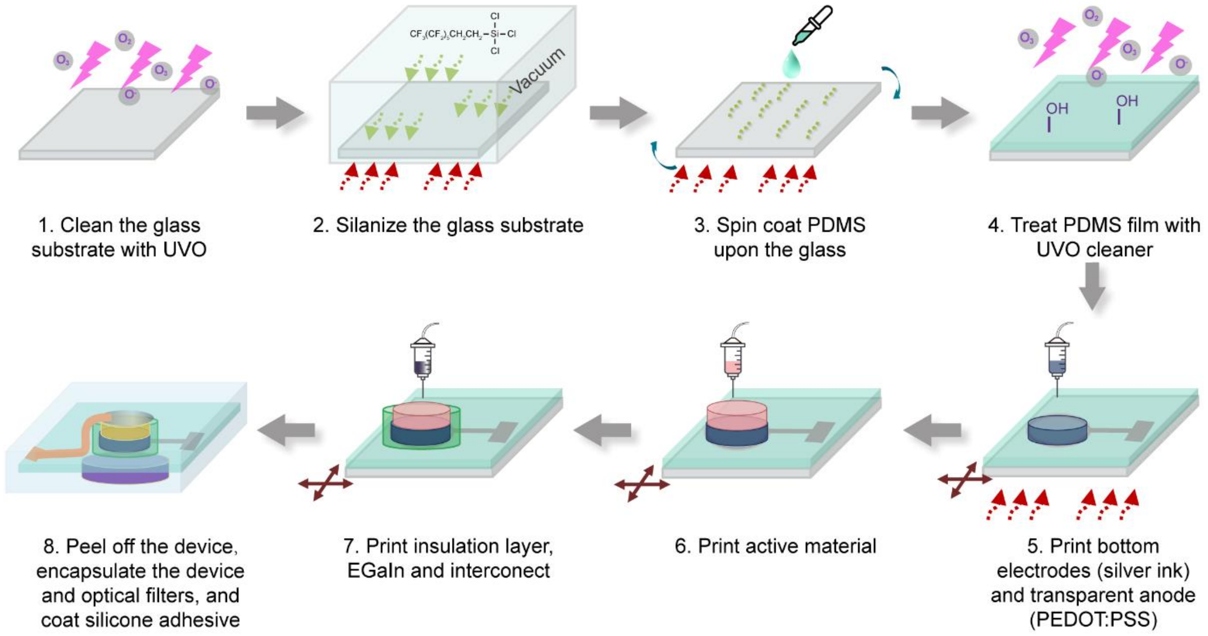

Figure S5. Process flow chart for 3D printing of photodetector.

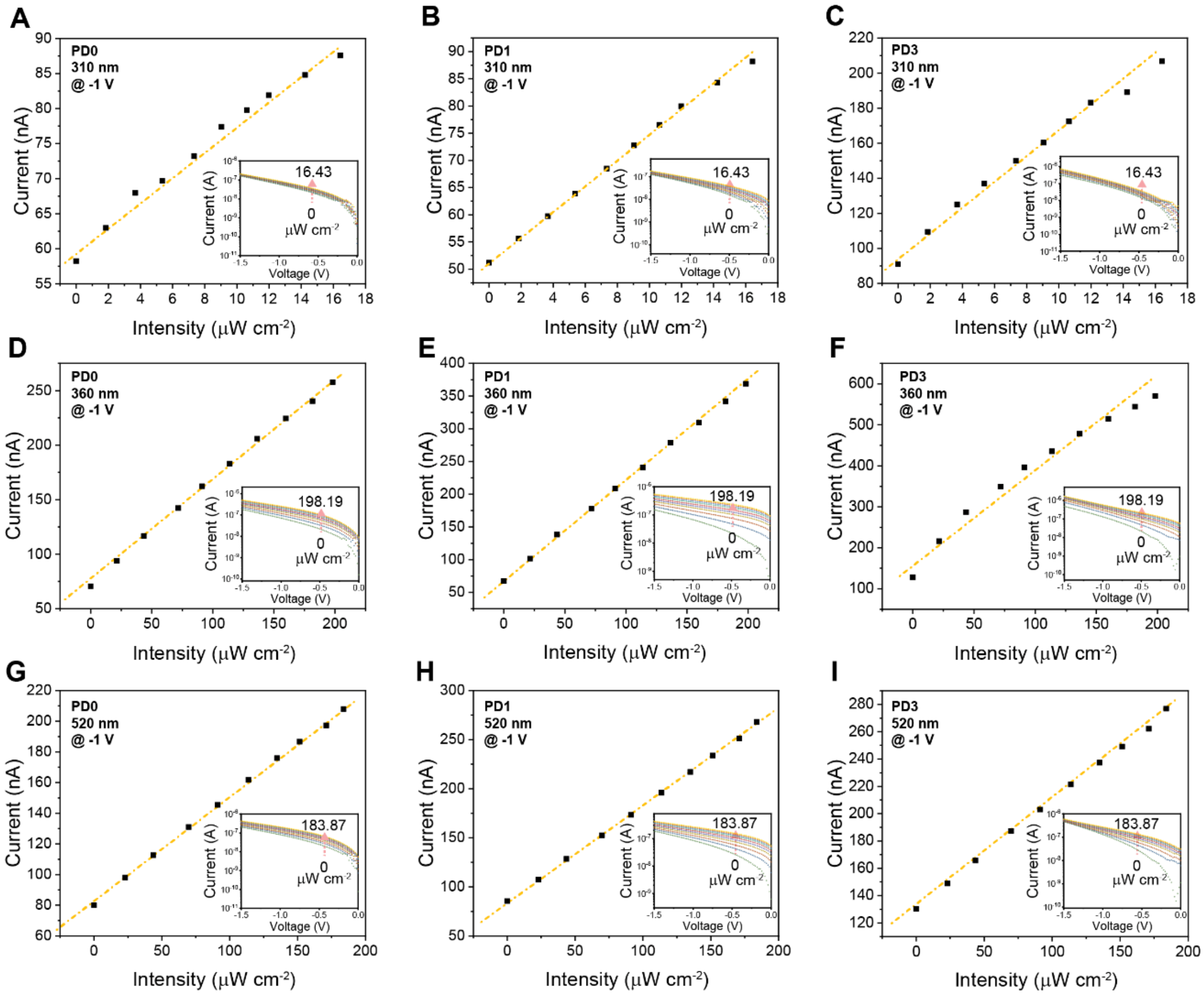

Figure S6. Characterization of the sensitivities of the photodetectors. A-C) Current as a function of light intensity (-1 V bias) at 310 nm of PD0, PD1, and PD3, respectively. D-F) Current as a function of light intensity (-1 V bias) at 360 nm of PD0, PD1, and PD3, respectively. G-I) Current as a function of light intensity (-1 V bias) at 520 nm of PD0, PD1, and PD3, respectively. The insets are current-voltage curves at varying light intensities.

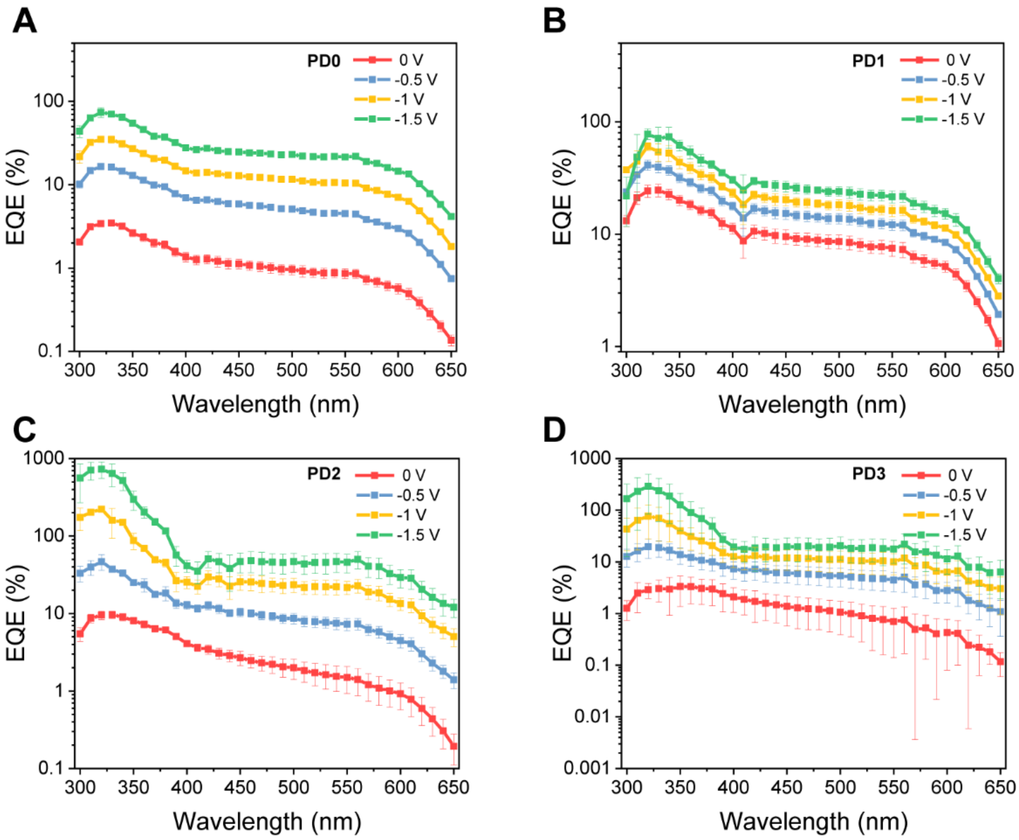

Figure S7. EQE of the photodetectors at varying bias voltages. EQE of A) PD0, B) PD1 C) PD2 and D) PD3, respectively (*n*=3). All data are presented as mean ± SD.

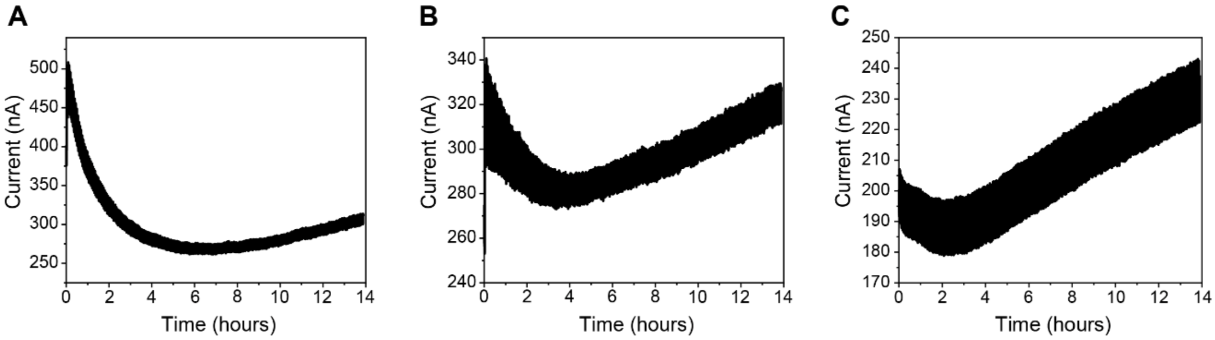

Figure S8. Long-term stability test. Current response of the photodetector to A) 310 nm, B) 360 nm and C) 520 nm on/off modulated light over a test period of 14 hours.

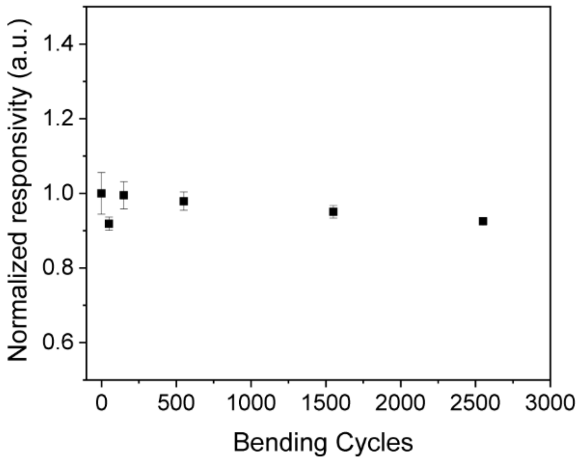

Figure S9. Responsivity stability during cyclic bending test (n=5). The bending curvature was 0.77 cm^{-1}. A 405-nm laser diode with a light intensity of 47.46 μW cm^{-2} and bias voltage of -1 V was used in the test. The plot is normalized to the responsivity of the photodetector at zero bending. All data are presented as mean ± SD.

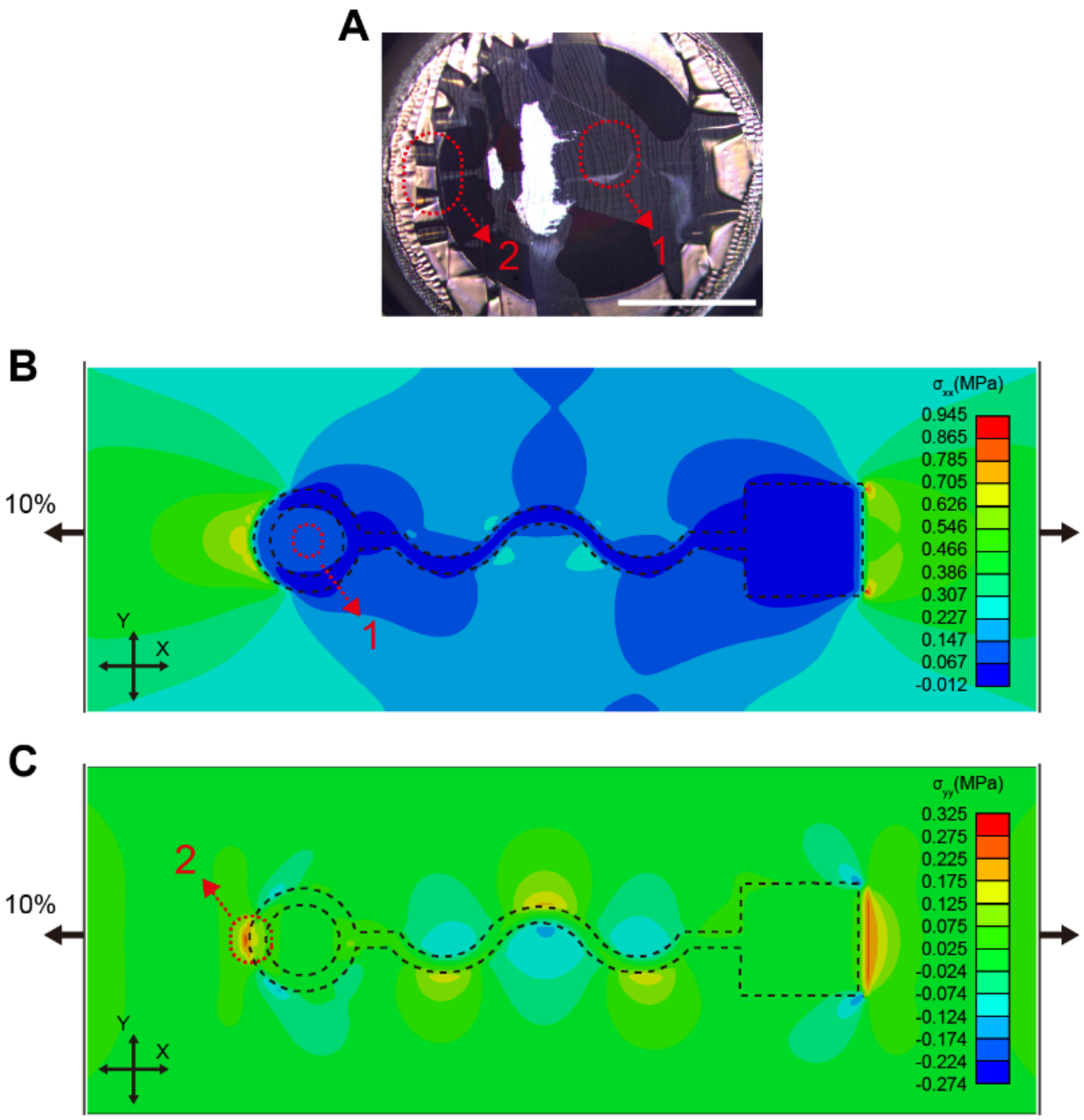

Figure S10. Analysis of the stress distribution of the PDMS substrate. A) Optical microscope image of photodetector after tensile test. The scale bar is 1 mm. B) and C) Stress distribution under 10% strain along B) *x*-axis and C) *y*-axis. The electrode is outlined by the dashed line.

Finite element analysis of photodetector

Finite element models of a PDMS layer and a silver electrode were developed to understand the experimentally observed failure of the device under tensile loading. The PDMS layer and silver electrode were discretized with 24,843 and 5,841 linear hexahedral elements with 8 nodes, respectively. The width (10 mm) and length (30 mm) of the PDMS layer were modeled to be large enough to avoid boundary effects. The geometry of the silver electrode matched the printed device. The thicknesses of the PDMS (155 µm) and silver electrode (2.14 µm) were modeled based on measurement results. A tied contact was used between the PDMS and electrode models. The PDMS layer and silver electrode were modeled as elastic solids. The

elastic modulus and Poisson's ratio of the PDMS layer were 2.61 MPa and 0.5, respectively.[S2] The elastic modulus and Poisson's ratio of the electrode were 83 GPa and 0.37,[S3] respectively. A finite element-predicted stress distribution of the PDMS layer was obtained under 10% strain, matching the experimental configuration. The PDMS and silver electrode were modeled in SOLIDWORKS 2021, and the finite element simulations were conducted in Abaqus.

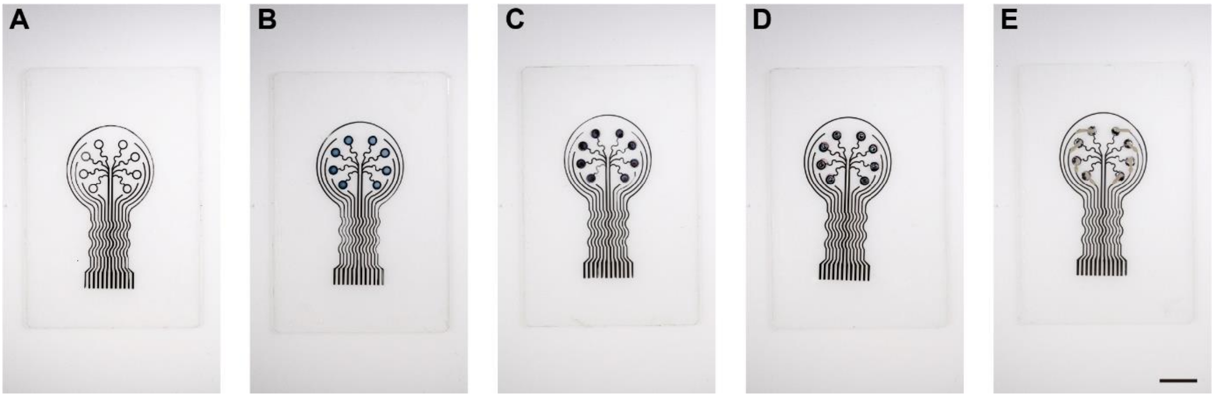

Figure S11. Fabrication steps of the photodetector array. A) Printing of electrodes on UVO-treated PDMS film with AgNPs. B) Printing of PEDOT:PSS transparent anode. C) Printing of active materials on transparent windows of electrodes. D) Printing of the silicone insulation layer and liquid metal cathode. E) Printing of top the conductive interconnects with silver paste. The scale bar is 10 mm.

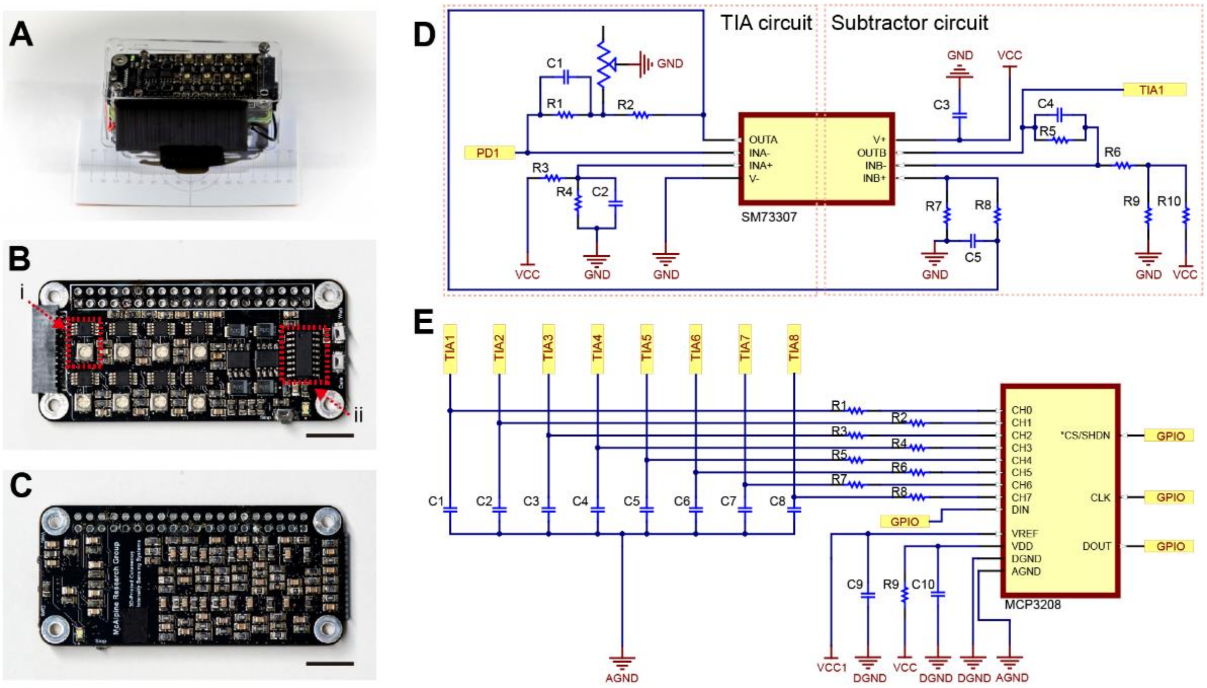

Figure S12. Custom-built signal processing board. A) The console for the photodetector array (back side view). B) and C) The front side and back side of the custom-built signal processing board in the console, respectively. The scale bars are 10 mm. The schematics of the circuits in B-i and B-ii are shown in D) and E), respectively.

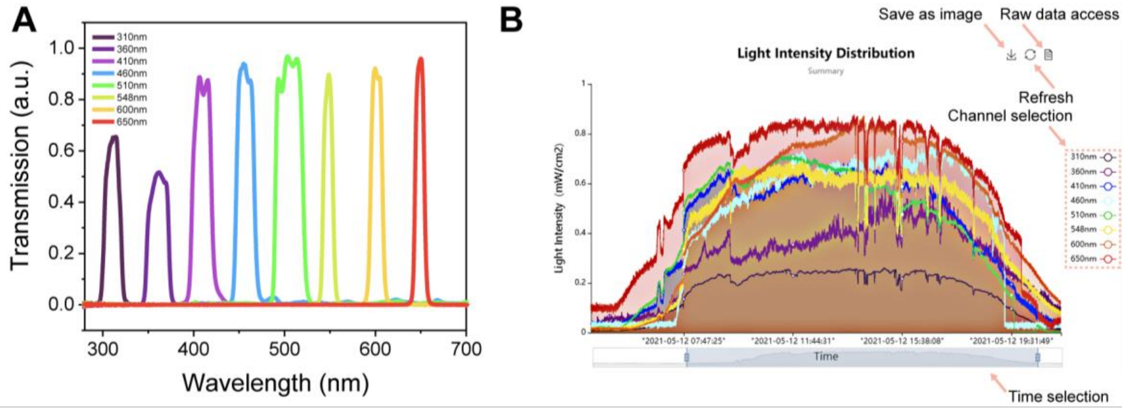

Figure S13. Characterization of the optical filters and demonstration of the web server. A) Transmission spectra of eight optical filters with varying central wavelengths in the photodetector array. B) Screenshot of the web server on the console. The server contains an interactive interface for time and channel selection and functions, including image generation, raw data access, and refreshing.

Table S1. Printing conditions for photodetectors

Inks	Nozzle Inner Diameter (μm)	Pressure (kPa)	Speed (mm/s)	Dispensing Time (s)	Curing Conditions
AgNP dispersion	80	1.2	10	-	130 °C, 30 min
PEDOT:PSS	100	1.1	0.8	-	130 °C, 15 min
Active material	100	0.3	-	1.2	-
RTV Silicone	100	1,400	1.5	-	Room temperature
EGaIn	200	100	-	0.03	-
Silver paste	250	100	0.5	-	Room temperature

Movie S1. Printing process of the photodetector array on the PDMS substrate. The silver electrodes, PEDOT:PSS transparent anodes, hybrid active materials, insulation layers and EGaIn cathodes are 3D printed on the UVO-treated PDMS film in a layer-by-layer process.

Movie S2. Skin-interfaced light intensity monitoring system. The encapsulated 3D printed photodetector is firmly attached to the arm via a silicone adhesive and shows water resistance.

Movie S3. Web server data of the light intensity monitoring system. The interactive graphical user interface of the web server provides multiple functions including channel (wavelength) selection, time period selection, raw data access, image generation, and data refreshing.

References

[S1] a) K. Matsumoto, K. Yamashita, Y. Sakoda, H. Ezoe, Y. Tanaka, T. Okazaki, M. Ohkita, S. Tanaka, Y. Aoki, D. Kiriya, S. Kashimura, M. Maekawa, T. Kuroda - Sowa, T. Okubo, *Eur. J. Org. Chem.* **2021**, *2021*, 4620; b) X. Zhang, E. Zheng, M. R. Esopi, C. Cai, Q. Yu, *ACS Appl. Mater. Interfaces* **2018**, *10*, 24064; c) S. H. Park, R. Su, J. Jeong, S. Z. Guo, K. Qiu, D. Joung, F. Meng, M. C. McAlpine, *Adv. Mater.* **2018**, *30*, 1803980.
[S2] a) Z. Wang, A. A. Volinsky, N. D. Gallant, *J. Appl. Polym. Sci.* **2014**, *131*, 41050; b) A. Muller, M. C. Wapler, U. Wallrabe, *Soft Matter* **2019**, *15*, 779.
[S3] E. K. McCarthy, A. T. Bellew, J. E. Sader, J. J. Boland, *Nature Commun.* **2014**, *5*, 4336.
